# Development of a Quality Assurance Score for the Nigeria AIDS Indicator and Impact Survey (NAIIS) Database: Validation Study

**DOI:** 10.2196/25752

**Published:** 2022-01-28

**Authors:** Hamisu M Salihu, Zenab Yusuf, Deepa Dongarwar, Sani H Aliyu, Rafeek A Yusuf, Muktar H Aliyu, Gambo Aliyu

**Affiliations:** 1 Center of Excellence in Health Equity, Training and Research Baylor College of Medicine Houston, TX United States; 2 Department of Family and Community Medicine Baylor College of Medicine Houston, TX United States; 3 Menninger Department of Psychiatry and Behavioral Sciences, Center for Innovations in Quality, Effectiveness and Safety, JP McGovern Campus Baylor College of Medicine Houston, TX United States; 4 Addenbrooke’s Hospital Cambridge United Kingdom; 5 Department of Management, Policy, and Community Health School of Public Health University of Texas Health Science Center at Houston Houston, TX United States; 6 Institute for Global Health Vanderbilt University Nashville, TN United States; 7 National Agency for the Control of AIDS Abuja Nigeria

**Keywords:** database quality assurance, Delphi method, quality assurance tool, Nigeria AIDS Indicator and Impact Survey

## Abstract

**Background:**

In 2018, Nigeria implemented the world’s largest HIV survey, the Nigeria AIDS Indicator and Impact Survey (NAIIS), with the overarching goal of obtaining more reliable metrics regarding the national scope of HIV epidemic control in Nigeria.

**Objective:**

This study aimed to (1) describe the processes involved in the development of a new database evaluation tool (Database Quality Assurance Score [dQAS]) and (2) assess the application of the dQAS in the evaluation and validation of the NAIIS database.

**Methods:**

The dQAS tool was created using an online, electronic Delphi (e-Delphi) methodology with the assistance of expert review panelists. Thematic categories were developed to form superordinate categories that grouped themes together. Subordinate categories were then created that decomposed themes for more specificity. A validation score using dQAS was employed to assess the technical performance of the NAIIS database.

**Results:**

The finalized dQAS tool was composed of 34 items, with a total score of 81. The tool had 2 sections: validation item section, which contains 5 subsections, and quality assessment score section, with a score of “1” for “Yes” to indicate that the performance measure item was present and “0” for “No” to indicate that the measure was absent. There were also additional scaling scores ranging from “0” to a maximum of “4” depending on the measure. The NAIIS database achieved 78 out of the maximum total score of 81, yielding an overall technical performance score of 96.3%, which placed it in the highest category denoted as “Exceptional.”

**Conclusions:**

This study showed the feasibility of remote internet-based collaboration for the development of dQAS—a tool to assess the validity of a locally created database infrastructure for a resource-limited setting. Using dQAS, the NAIIS database was found to be valid, reliable, and a valuable source of data for future population-based, HIV-related studies.

## Introduction

HIV continues to be a global major public health threat, with about 38 million people living with the disease as of 2018 [1]. Nigeria, Africa’s most populous country, with an estimated population of 203 million is home to 1.9 million individuals living with HIV/AIDS, making it the nation with the fourth highest number of individuals living with HIV/AIDS [1,2]. In addition, Nigeria ranks among the top 6 nations in the world that bear the triple threat of high HIV infection, low treatment coverage, and slow decline in new HIV infections [3]. Against this background, it becomes necessary to have high-quality, reliable, accurate, and timely public health information for improving, evaluating, and monitoring HIV-related health care services and programs [4,5]. However, resource-limited settings like Nigeria are continuously challenged by low-quality data that are often incomplete, unreliable, and inaccurate, which blunt their versatility for decision-making [4,5]. Data quality audits play a significant role in assessing if data meet the quality mandated to support their proposed use [6].

In 2017, Nigeria launched the Nigerian AIDS Indicator and Impact survey (NAIIS) with the overarching goal of obtaining reliable population-based metrics regarding the scope of the HIV situation in Nigeria. The NAIIS is a multistakeholder endeavor to reliably estimate the scope and burden of HIV in Nigeria to enable policy makers and stakeholders to address gaps in access to care, linkage to care and retention, treatment coverage, and viral RNA suppression. The NAIIS project gathered comprehensive information on sociobehavioral attributes, linkage to care, levels of HIV viral load suppression (VLS), hepatitis B and hepatitis C coinfection, and other important data. The NAIIS is the largest population-based HIV survey ever undertaken to date. The NAIIS is also the first population-based study to include VLS, pediatric HIV prevalence, and antiretroviral therapy coverage as outcome assessments. The survey and estimates of biomarkers will provide critical data to assess the Joint United Nations Programme on HIV/AIDS (UNAIDS) 95/95/95 treatment targets at a national level.

The richness and complexity of the NAIIS project were reflected by the vast resources infused as well as the array of stakeholders involved in its planning, implementation, and monitoring. It was a joint endeavor of the Government of Nigeria (GON); Federal Ministry of Health; National Agency for the Control of AIDS (NACA); the US Government President’s Emergency Plan for AIDS Relief (PEPFAR) program in Nigeria; the US Centers for Disease Control and Prevention (CDC) in Nigeria and Atlanta; implementing partners from the University of Maryland, Baltimore (UMB); and data management partners, ICF Macro, as well as the Institute for Health Metrics and Evaluation (IHME). ICF Macro worked in partnership with UMB in the implementation of the survey to support data management. In this paper, we describe primarily the processes involved in the development of a new database evaluation tool called the Database Quality Assurance Score (dQAS) and secondarily the use of the dQAS to evaluate and validate the NAIIS database emanating from the multistakeholder project.

## Methods

**Figure 1:**
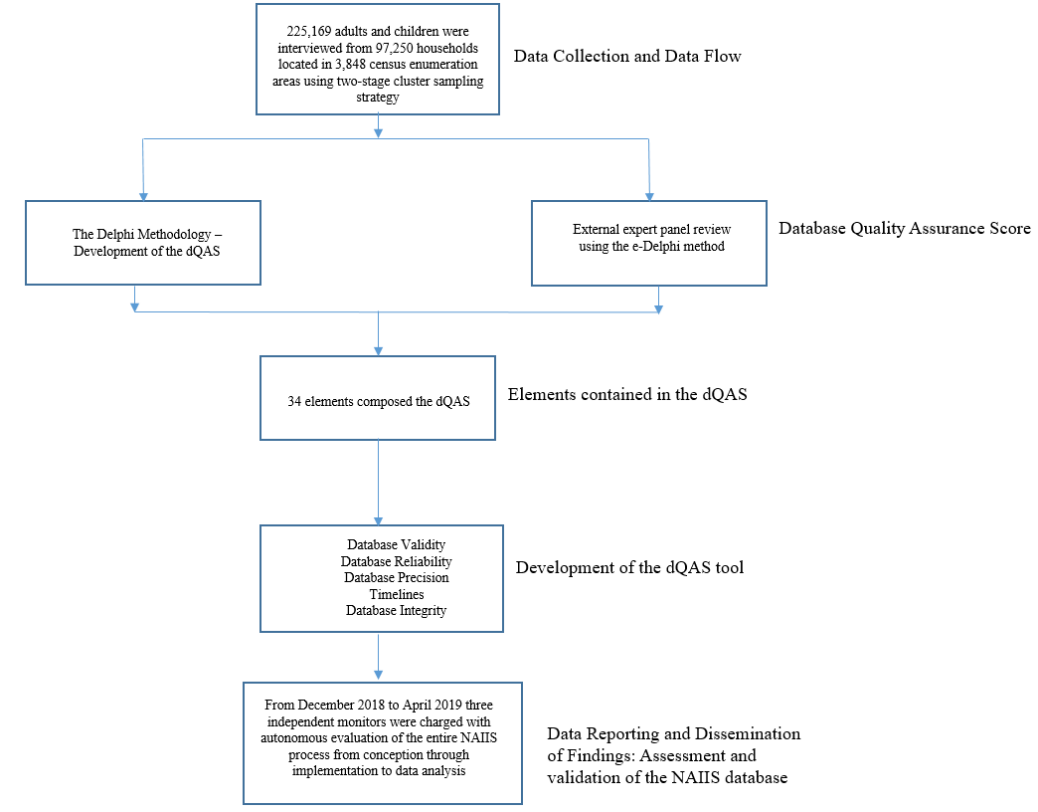
Flowchart showing the steps and processes. dQAS: Database Quality Assurance Score; e-Delphi: electronic Delphi; NAIIS: Nigeria AIDS Indicator and Impact Survey.

The flow of the steps and processes followed in this project are shown in Figure 1.

### Data Collection and Data Flow

From July 2018 to December 2018, 225,169 adults and children were interviewed from 97,250 households located in 3848 census enumeration areas in this 2-stage cluster sampling cross-sectional study. Over 6000 field staff worked for 22 consecutive weeks to conduct interviews, collect blood samples, and perform rapid immunologic tests. Questionnaire and field laboratory data (eg, rapid test results) were collected on mobile tablet devices using the Census and Survey Processing System (CSPro; a data capture software developed by the US Census Bureau). Within the household, questionnaire and laboratory data were transmitted between tablets via Bluetooth connections. Team leads transmitted all survey data collected in CSPro via FTPS over a 4G or 3G telecommunications provider at least once a day. We used https via a 4G or 3G telecommunications provider for transmission of data to the central server by the field, laboratory, and logistics teams. Survey data as well as field laboratory data were synchronized daily to the main server. In addition to the server transfer, daily backups were made to secure portable USB drives that were stored in a different location. Paper tools were used to monitor daily data collection activities. There were 18 data entry persons that worked over the course of 150 days entering data from the field, yielding a total of 2700 data entry personnel days. On average, 1380 individual interview records were entered per day from the 18 data entry personnel, resulting in a total of 207,000 individual record entries over the course of the survey. An activity information management system was used to centrally manage all the data collected. These included specimen results and location data captured in a laboratory data management system, interview data captured in CSPro, SureMDM data, procurement and inventory management data, and personnel data.

### Database Quality Assurance Score

#### The Delphi Methodology—Development of the dQAS

Initially, we created an internal review panel comprised of 6 members and 1 facilitator based on their expertise in the following areas: (1) database management, (2) data science, and (3) epidemiology. These internal reviewers were charged with an initial assessment of metrics abstracted from the literature and the World Health Organization (WHO) guidelines for database quality evaluation [7]. The reviewers assessed domain coverage and candidate metrics by analyzing illustrative metrics items from the WHO guidelines and the published literature and identifying poorly informative database quality metrics, which were discarded or altered. Our team of experts then examined the potential candidate metrics pool with the purpose of creating the dQAS.

First, each reviewer assessed database evaluation domain coverage based on the following criteria: (1) relevance to practical application, (2) measurable within the developing country context, (3) clarity, and (4) conciseness. Each criterion was applied using a 7-point Likert-type rating scale (1=Poor to 7=Excellent). After rating each candidate metric on the 4 criteria, the ratings for each metric were averaged. Subsequently, each reviewer ranked the pool of itemized metrics. Top-ranked items were then shared and discussed in a plenary session. Consensus was attained when 2 reviewers independently selected the metric. An additional reviewer was consulted for a final decision on highly scored items (eg, more than 20 points) that were not selected by the initial reviewers. Following this preselection process, we were left with a manageable list of metrics of less than 100 items. By using this systematic approach, our team was able to exclude redundant items that did not have sufficient face validity for use in the development of a database assessment instrument. All selected items were entered in an item library with documentation of their definition and domain coverage.

#### External Expert Panel Review Using the Electronic Delphi Method

To enhance the validity of our internal panel review, we submitted the list of preselected metrics items to a sample of 8 database development and evaluation experts as well as community stakeholders outside the research team to gather diverse input and establish consensus regarding candidate metrics. They were asked to apply the same set of rating criteria used by the research team but to a manageable list of preselected items, with the goal of verifying the relevance of the metrics in measuring key aspects of database quality assessment. For this purpose, we used a technology-enhanced Delphi method using online data collection [8]. The Delphi method is a well-established technique of gathering opinions from a diverse group of experts and is particularly useful for forecasting and decision-making on practice-related issues [8]. This technique produces reliable expert panel consensus through iterative rounds of questions while encouraging open feedback and maintaining anonymity and confidentiality. Also, the Delphi technique is preferable to the nominal group technique or focus groups when the purpose is to generate more stable estimates that are comparable to statisticized groups [9,10].

The Delphi technique consisted of iterative sequential rounds of questions with experts, which was implemented online. The online modality (electronic Delphi [e-Delphi]) was preferred because it permitted an efficient and relatively quicker assessment of expert panel consensus [11]. During the first round of questions, all preselected metrics were presented to each expert in an anonymous online survey created and distributed via email with Qualtrics software [12]. In a second round of questions, the group results were presented online using a secure link that assured anonymity and confidentiality of responses. In this round, panelists had the opportunity to reconsider their answers based on the aggregated data. Ratings were then analyzed quantitatively using the median as a cut-off point, and items rated higher than or equal to the group median were included for subsequent phases of the study [13,14]. Results were presented in a subsequent round, highlighting areas of agreement and disagreement, and opportunities for open-ended comments were offered. The final result was a set of database quality assurance metrics based on a robust expert-driven review process incorporating multifaceted perspectives.

### Elements Contained in the dQAS

The dQAS contained the elements that are listed in Multimedia Appendix 1.

### Development of the dQAS Tool

A thematic framework was created and adapted for this study using the United States Agency for International Development (USAID) data quality assessments checklist derived from the USAID’s Automated Directives System Chapter 597 Operations Performance Policy [15,16]. Thematic categories were developed to form superordinate categories that grouped themes together. Additionally, subordinate categories were created that decomposed themes for more specificity. This culminated in the development of the dQAS Tool (Multimedia Appendix 2).

### Data Reporting and Dissemination of Findings: Assessment and Validation of the NAIIS Database

From December 2018 to April 2019, 3 independent monitors were charged with autonomous evaluation of the entire NAIIS process from conception through implementation to data analysis. One of the tasks of the project monitors was to utilize the dQAS tool built for the NAIIS project to validate the database.

## Results

### The dQAS Tool

The dQAS tool is a qualitative key database performance measure consisting of 34 items with a total score of 81. The dQAS tool has 2 sections: validation item section and quality assessment score section. The validation item section is further categorized into 5 subsections (Table 1). These include (1) database validity (20 items), (2) database reliability (5 items), (3) database precision (4 items), (4) timeliness (1 item), and (5) database integrity (5 items). The quality assessment score section was assigned a score of “1” for “Yes” (performance measure item present) and “0” for “No” (measure absent). There were also additional scaling scores ranging from “0” to a maximum of “4” depending on the measure (Multimedia Appendix 1 and Multimedia Appendix 2).

**Table 1:** Outcomes of implementing the Database Quality Assurance Score (dQAS) tool on the Nigeria AIDS Indicator and Impact Survey (NAIIS) database.

Superordinate and subordinate categories (number of elements)			Quality assessment score
**Data validity (subtotal=37)**			
	Data-entry-sample ratio (DESR)	2	
	Data entry/management personnel training	1	
	Data entry/management personnel certification	1	
	Data entry/management personnel troubleshooting session	1	
	Frequency of troubleshooting sessions	3	
	Presence of a data management supervisor	1	
	Presence of a data management deputy supervisor	1	
	Qualification of data managers	3	
	Qualification of the data manager supervisor	3	
	Data entry personnel/manager’s database knowledge assessment	3	
	Type of database	1	
	Database selection justification	1	
	Architecture of database corresponding with the working conceptual framework	1	
	Database degree of complexity	1	
	Concordance of prevalence estimates	5	
	Weighting algorithm consideration	1	
	Justification of the weighting process	3	
	Appropriateness of the weighting algorithm	3	
	Files backup and transfer systems	1	
	Database dictionary creation	1	
**Database reliability (subtotal=5)**			
	Presence of data audit system	1	
	Presence of in-built checks mechanism	1	
	Presence of alert or inactivation system	1	
	Presence of additional audit systems	1	
	Employment of a double key data entry validation process	1	
**Database precision (subtotal=9)**			
	Variable missing ratio (VMR)	4	
	Observation missing ratio (OMR)	4	
	Duplicate ratio	1	
**Timeliness (subtotal=8)**			
	Quality assessment of the database dictionary	8	
**Database integrity (subtotal=19)**			
	Presence of a data and safety monitoring board (DSMB)	1	
	Description of DSMB membership and expertise of members	3	
	Database security and risk management procedures	12	
	Presence of external independent monitors	1	
	Database Transparency Index (DTI)	2	

### Qualitative Findings of the Survey Evaluation

The NAIIS Evaluation Instrument score results are shown in Table 1.

The dQAS tool results from the NAIIS are summarized in Table 2. The maximum score for each validation item section was assessed as 38 for database validity, 5 for the database reliability, 9 for timeliness, and 21 for database integrity (Table 2). The maximum total score was 81, out of which the NAIIS database achieved 78, or 96.3%, which placed it in the highest category denoted as “Exceptional.”

**Table 2:** Database Quality Assurance Score (dQAS) categories, elements, and scores.

Validation item (superordinate categories)	Subordinate categories (number of elements)	Quality assessment score (subtotal=81)			Database assessment score (subtotal=78)
		Minimum	Maximum		
Data validity	20	0	38	37	
Database reliability	5	0	5	5	
Database precision	4	0	9	9	
Timeliness	1	0	8	8	
Database integrity	5	0	21	19	

There were 2 areas in which the NAIIS database scored less than the maximum score. The first was the data-entry-sample ratio (DESR), which captured the number of data management persons per 1000 samples per day and was calculated as the proportion of personnel per daily data waves: the larger the proportion, the greater the personnel adequacy and the lower the expected error rate. The maximum score on this metric was 3 points. However, the NAIIS database achieved a score of 2 because its DESR was 13/1000 samples. The NAIIS database also scored suboptimally on the Database Transparency Index (DTI), which measured the extent to which independent accessors had access to the database. The NAIIS database scored 2 out of a maximum of 4 points on this index. Multimedia Appendix 3 provides the details of the NAIIS database system evaluation findings.

The overall dQAS was categorized as Exceptional (≥95%; score of 77 out of 81), Outstanding (90%-94%; score of 73-76 out of 81), Excellent (85%-89%; score of 69-72 out of 81), Very Good (80%-84%; score of 65-69 out of 81), Good (75%-79%; score of 61-64 out of 81), Fair (65%-74%; score of 61-64 out of 81), or Poor (≤64%; score of ≤60 out of 81).

## Discussion

We evaluated and validated the NAIIS database derived from the world’s largest population-based HIV survey conducted in Nigeria. We achieved this by using the e-Delphi method to develop the dQAS tool. We then applied the derived dQAS tool to assess the quality of the NAIIS database, which attained an overall exceptional score of 96.3% (score of 71 out of 81). Hence, we found the database to be valid for future scientific and scholarly work that would advance the field.

In our study, we utilized an e-Delphi method that was implemented via the internet, which enabled obtaining input from panelists who were living in different parts of the world. Compared with the traditional format, the e-Delphi method minimized prolonged delays in arriving at consensus and decreased nonparticipation by expert panel members associated with the traditional Delphi technique, which typically employs surface or airmail systems to ensure anonymity [8,17,18]. Additionally, the e-Delphi method was flexible, convenient, and cost-effective and allowed for more robust collaboration between local and international researchers in our study [17-20].

An added quality assurance interface in our study that enhanced the rigor of the assessment was the layering of the e-Delphi into an internal and an external expert review panel. The internal expert review panelists performed the preselection of metrics for the creation of the dQAS. In addition, they eased the subsequent work by the external expert review panelists who were not directly involved in the study and whose main role was to authenticate candidate metrics. This 2-stage expert consensus-driven process facilitated by the internet-dependent e-Delphi method ensured reliability and validity of the metrics for developing dQAS, in addition to assuring that our study was conducted more effectively and efficiently [8,11,18,20].

There are multiple strengths in this study. A merit of the methodology is that it showed the feasibility of remote internet-based collaboration for the development of a tool to assess the validity of a database infrastructure. We specifically illustrated a feasible North-South partnership to establish the validity of a database that was locally created in a low-middle-income country. This broader approach enhances the robustness of the process. As with any study, there were certain limitations. We believe that more access should have been offered to the database evaluators, including running some of the analyses themselves. However, database managers were concerned about the likelihood of breaching the confidentiality agreement signed with the GON. Consequently, the DTI was assigned a very low score of 2 out of a maximum of 4. Despite this deficit, the overall technical performance of the database on the dQAS instrument was exceptional (71 out of 81, 96.3%). In conclusion, the NAIIS database is valid and reliable and has been proven to be a useful data source for future research projects. Further, the dQAS represents a unique database assessment asset that could be utilized by other countries, with modifications as needed.

## Appendix

Multimedia Appendix 1 Database Quality Assurance Score (dQAS).

Multimedia Appendix 2 Database Quality Assurance Score (dQAS) tool.

Multimedia Appendix 3 Database management system: Database Quality Assurance Score (dQAS).

## Abbreviations

CDC: Centers for Disease Control and Prevention
CSPro: Census and Survey Processing System,
DESR: data-entry-sample ratio
dQAS: Database Quality Assurance Score
DTI: Database Transparency Index
e-Delphi: electronic Delphi
FMOH: Federal Ministry of Health
GON: Government of Nigeria
IHME: Institute for Health Metrics and Evaluation
LDMS: laboratory data management system
NACA: National Agency for the Control of AIDS
NAIIS: Nigeria AIDS Indicator and Impact Survey
PEPFAR: President’s Emergency Plan for AIDS Relief
UMB: University of Maryland, Baltimore
UNAIDS: Joint United Nations Programme on HIV/AIDS
USAID: United States Agency for International Development
VLS: viral load suppression
WHO: World Health Organization
