# Supplementary material for: Development of a Quality Assurance Score for the Nigeria AIDS Indicator and Impact Survey (NAIIS) Database: Validation Study
Source: JMIR Form Res. 2022 Jan 28;6(1):e25752. doi: 10.2196/25752 (PMC8838544; doi:10.2196/25752)
Supplement: Multimedia Appendix 1 [file formative_v6i1e25752_app1.docx]

**Appendix A.**

*Database Quality Assurance Score (dQAS)*

1. What *type* of database is being used and assessment of its specification
    Appears adequate

Appears inadequate

1. Assessment of database knowledge among a sample of data entry personnel/managers:
   1. Could name the database

Yes
No

- 1. Could describe what each database platform does

Yes
No

- 1. Know how to detect errors even before data entry by giving an example of such an error

Yes
No

1. Any justification for the selection of the database?

Yes
No

1. Does the architecture of the database correspond to the working conceptual framework?

Yes
No

1. Degree of complexity: Is it a *two-generation* database (e.g., mother, child, father captured and could be linked)?

Yes
No

1. Was *training* provided for data entry/management personnel?

Yes
No

1. Is there any *certification* required for the data entry/management personnel before placement on the field?

Yes
No

1. Presence of a *data management supervisor*?

Yes
No

1. Presence of a *data management deputy supervisor*?

Yes
No

1. *Qualification of Data Manager*. Done for each data manager

Less than high school

High school

First degree

Post-graduate

1. *Qualification of Data Manager Supervisor*

Less than high school

High school

First degree

Post-graduate

1. Is there a regular trouble-shooting session for data entry/management team?

Yes
No

1. Frequency of trouble-shooting sessions

None

one per ≥ 28 days
one per 7-28 days
one per ≤ one day

1. *Data-entry-sample ratio* *(DESR)*
   <5
   5-9
   10-14
   ≥15
2. *Concordance of prevalence estimates* for selected variables and comparison with expected or projected estimates. Grand Mean Difference of :

<5%

5-9%

10-19%

20-29%

30-39%

≥40%

1. Was a weighting algorithm considered?

Yes
No

1. Justification of the weighting process is clearly stated or specified

Unclear

Somewhat clear

Clear

Clear with compelling justification

1. Appropriateness of the weighting algorithm

Inappropriate

Somewhat appropriate

Appropriate

Highly appropriate

1. Files backup and transfer systems

Yes
No

1. Is there a database/dataset *dictionary* created?

Yes
No

1. Presence of *data audit* system? E.g., regular sampling of the data and assessment for errors.

Yes
No

1. Presence of *in-built checks mechanism* (e.g., rejection of character where numerical values are specified and vice versa).

Yes
No

1. Presence of alert or inactivation system based on information non-concurrence (e.g., gender is male but the pregnancy space is filled out in the affirmative).

Yes
No

1. Presence of *additional* audit systems.

Yes
No

1. Does the database system in place employ a double key data entry validation process?

Yes
No

1. *Variable Missing Ratio (VMR)*

0% missing

1-9% missing

10-19% missing

20-29%

≥30%

1. *Observation Missing Ratio* (*OMR*)

0% missing

1-9% missing

10-19% missing

20-29%

≥30%

1. *Duplicates ratio*: total number of duplicates divided by total number of unique records. The lower the proportion the better the data quality:
2. *Quality assessment* of the database dictionary:
   1. Logical *coding language* (e.g., child_age rather than age_2)

Yes
No

- 1. *Each coded variable* is described clearly and is self-explanatory.

Yes
No

- 1. *Synonymous variables* (those measuring the same attribute) are identified and explained.

Yes
No

- 1. *Value for each variable* correctly coded and consistent (e.g., age in years throughout and NOT in years and months for some records).

Yes
No

- 1. *User instructions* on how to employ the database/dataset are available.

Yes
No

- 1. *Database/dataset dictionary* is accessible online with a search function capability.

Yes
No

- 1. Available *automatic updating system* (e.g., modification of a variable in the database is automatically reflected in the dictionary)

Yes
No

- 1. Is there *a blinded data generation and reconciliation mechanism* in place (e.g., two anonymous statisticians produce results of a task independently and discrepancy addressed. This needs to be tested real-time)?

Yes
No

1. Presence of *Data and Safety Monitoring Board* (*DSMB*) to periodically evaluate accumulated data and ascertain data security.

Yes
No

1. If a form of *DSMB* exists, describe the membership and expertise of members of this or its equivalence.

All members are from same background (e.g., all are statisticians)

All members are from less than three professional/academic background

All members are from ≥ 3 professional backgrounds

1. *Database* *security and risk management procedures*: System in place to ensure the following (this needs to be verified):
   1. Prevention of *unauthorized intrusion*

Yes
No

- 1. *Audit trails* of all activities by staff and administrators

Yes
No

- 1. *Database replication and synchronization*: replication of database to other servers

Yes
No

- 1. Well-defined *compartments of privileges*

Yes
No

- 1. *Coding review layering* (how many verification layers ascertain accuracy of codes?).

None

Single layer (one reviewer)

Multiple layers (≥2 reviewers)

- 1. Periodic database *vulnerability testin*g (e.g., automated vulnerability scans to uncover database defects).

Yes
No

- 1. *Compliance monitoring* to ascertain security standards are observed

Yes
No

- 1. *DAM* (Database activity monitoring): e.g., through analysis of protocol traffic or observing local database activity on each server

Yes
No

- 1. *Separation of dutie*s between DAM and database administrators

Yes
No

- 1. *A two-factor authentication* system

Yes
No

- 1. *Control system* to prevent physical damage (e.g., from outage and extreme heat or power fluctuations)

Yes
No

1. Presence of external independent monitors or database assessors that are not part of the stakeholders or any structure of the study

None

At least one external assessor

1. *Database Transparency Index (DTI)*:

None

Fair

Good

Excellent
Outstanding
