# Supplementary material for: Development of a Quality Assurance Score for the Nigeria AIDS Indicator and Impact Survey (NAIIS) Database: Validation Study
Source: JMIR Form Res. 2022 Jan 28;6(1):e25752. doi: 10.2196/25752 (PMC8838544; doi:10.2196/25752)
Supplement: Multimedia Appendix 2 [file formative_v6i1e25752_app2.docx]

**Appendix B.

The Database Quality Assurance Score (dQAS) Tool**

| **Serial Number** | **Validation Item** | **Quality Assessment Score** | |
| --- | --- | --- | --- |
| **DATABASE VALIDITY** | | | |
|  | What type of database is being used, and assessment of its specification | 1= appears adequate | 0=appears inadequate |
|  | Assessment of database knowledge among a sample of data entry personnel/managers:  a. Could name the database  b. Could describe what each database platform does  c. Know how to detect errors even before data entry by giving an example of such an error | Yes=1 Yes=1  Yes=1 | No=0 No=0  No=0 |
|  | Any justification for the selection of the database? | Yes=1 | No=0 |
|  | Does the architecture of the database correspond to the working conceptual framework? | Yes=1 | No=0 |
|  | Degree of complexity: E.g., is it a two-generation database (e.g., mother, child, father captured and could be linked)? | Yes=1 | No=0 |
|  | Was training provided for data entry/management personnel? | Yes=1 | No=0 |
|  | Is there any certification required for the data entry/management personnel before placement on the field? | Yes=1 | No=0 |
|  | Presence of a data management supervisor | Yes=1 | No=0 |
|  | Presence of a data management deputy supervisor | Yes=1 | No=0 |
|  | Qualification of Data Managers | Less than high school= 0  High school = 1  First degree =2  Post-graduate = 3 |  |
|  | Qualification of Data Manager Supervisor | Less than high school =0  High school = 1  First degree =2  Post-graduate = 3 |  |
|  | Is there a regular trouble-shooting session for the data entry/management team? | Yes=1 | No=0 |
|  | Frequency of trouble-shooting sessions | None = 0  One per ≥ 28 days =1  One per 7-28 days =2  One per ≤ one day = 3 |  |
|  | Data-entry-sample ratio (DESR) = (the number of data management persons per 1000 samples per day). This is obtained by deriving the proportion of personnel per daily data waves. The greater the proportion the greater personnel adequacy and the lower the expected error rate. | <5 = 0  5-9 =1  10-14 = 2  ≥15 = 3 |  |
|  | *Concordance of prevalence estimates for selected variables and comparison with expected or projected estimates. | Grand Mean Difference of <5% = 5  5-9% =4  10-19% =3  20-29% =2  30-39% =1  ≥40 =0 |  |
|  | Was a weighting algorithm considered | Yes=1 | No=0 |
|  | Justification of the weighting process is clearly stated or specified. | Unclear = 0  Somewhat clear = 1  Clear =2  Clear with compelling justification = 3 |  |
|  | Appropriateness of the Weighting algorithm | Inappropriate = 0  Somewhat appropriate = 1  Appropriate =2  Highly appropriate = 3 |  |
|  | Files backup and transfer systems | Yes=1 | No=0 |
|  | Is there a database dictionary created? | Yes=1 | No=0 |
| **DATABASE RELIABILITY** | | | |
|  | Presence of data audit system? E.g., regular sampling of the data and assessment for errors | Yes=1 | No=0 |
|  | Presence of in-built checks mechanism (e.g., rejection of character where numerical values are specified and vice versa): | Yes=1 | No=0 |
|  | Presence of alert or inactivation system based on information non-concurrence (e.g., gender is male but the pregnancy space is filled out in the affirmative) | Yes=1 | No=0 |
|  | Presence of additional audit systems | Yes=1 | No=0 |
|  | Does the database system in place employ a double key data entry validation process? | Yes=1 | No=0 |
| **DATABASE PRECISION** | | | |
|  | Variable Missing Ratio (VMR) = [(number of variables with more than 10% missing data ÷ total number of variables) x 100]. The lower the proportion the better the data validity. | 0% to <1% missing = 4  1-9% missing = 3  10-19% missing = 2  20-29% =1  ≥30% =0 |  |
|  | Observation Missing Ratio (OMR) = [(total number of observations with more than 10% of the variables with missing information ÷ total number of observations) x 100]: The lower the proportion the better the data validity. | 0% to <1% missing = 4  1-9% missing = 3  10-19% missing = 2  20-29% =1  ≥30% =0 |  |
|  | Duplicates ratio: total number of duplicates divided by total number of unique records. The lower the proportion the better the data quality. | Yes=1 | No=0 |
| **TIMELINESS** | | | |
|  | Quality assessment of the database dictionary  a. Logical and coding language (e.g., child_age rather than age_2)  b. Each coded variable is described clearly and is self-explanatory  c. Synonymous variables (those measuring the same attributes) are identified and explained d. Value for each variable correctly coded and consistent (e.g., age in years throughout and NOT in years and months for some records)  e. User instructions on how to utilize the database/dataset are available  f. Database dictionary accessible online with a search function capability  g. Available automatic updating system (e.g., modification of a variable in the database is automatically reflected in the dictionary)  h. Is there a blinded data generation and reconciliation mechanism in place (e.g., two anonymous statisticians produce results of a task independently and discrepancy addressed)? | Yes=1  Yes=1  Yes=1  Yes=1  Yes=1  Yes=1  Yes=1  Yes=1 | No=0  No=0  No=0  No=0  No=0  No=0  No=0  No=0 |
| **DATABASE INTERGRITY** | | | |
|  | Presence of Data and Safety Monitoring Board (DSMB) to periodically evaluate accumulated data and ascertain data security | Yes=1 | No=0 |
|  | If a form of DSMB exists, describe the membership and expertise of members of this or its equivalence | All are from same background =1  All are from <3 professional backgrounds=2 All are from ≥ 3 professional backgrounds=3 |  |
|  | Database security and risk management procedures: System is in place to ensure the following:  a. Prevention of unauthorized intrusion  b. Audit trails of all activities by staff and administrators  c. Database replication and synchronization: replication of database to other servers  d. Well-defined compartments of privileges  e. Periodic database vulnerability testing (e.g., automated vulnerability scans to uncover database defects)  f. Compliance monitoring to ascertain security standards are observed  g. DAM (Database activity monitoring): e.g., through analysis of protocol traffic or observing local database activity on each server  h. Separation of duties between DAM and database administrators  i. A two-factor authentication system  j. Control system to prevent physical damage (e.g., from outage and extreme heat or power fluctuations)  k. Coding review layering (how many verification layers ascertain accuracy of codes | Yes=1  Yes=1  Yes=1  Yes=1  Yes=1  Yes=1  Yes=1    Yes=1    Yes=1  Yes=1  None =0  Single layer (one reviewer) = 1  Multiple layers (≥2 reviewers) = 2 | No=0  No=0  No=0  No=0  No=0  No=0  No=0    No=0    No=0  No=0 |
|  | Presence of external independent monitors or database assessors that are not part of the stakeholders or any structure of the study | 1= at least one external assessor | 0 = none |
|  | Database Transparency Index (DTI): To what extent are independent monitors allowed to have access to the auditing process of the database? This is subjectively evaluated based on the perception of the independent monitor/assessor. | None=0  Fair=1  Good=2  Excellent=3  Outstanding=4 |  |

***Source:** The United Nations Population Division's World Urbanization Prospects, 2016. Accessed @ <https://www.indexmundi.com/facts/nigeria/indicator/SP.URB.TOTL.IN.ZS> on 04/07/2019
