# Supplementary material for: Development of a Quality Assurance Score for the Nigeria AIDS Indicator and Impact Survey (NAIIS) Database: Validation Study
Source: JMIR Form Res. 2022 Jan 28;6(1):e25752. doi: 10.2196/25752 (PMC8838544; doi:10.2196/25752)
Supplement: Multimedia Appendix 3 [file formative_v6i1e25752_app3.docx]

**Appendix C: Database Management System**: **Database Quality Assurance Score (dQAS)**

| **Validation Item** | **Maximum Quality Assessment Score** | **Comment** |
| --- | --- | --- |
| Database Infrastructure |  |  |
| What type of database is being used, and assessment of its specification | 1 | The CSPro (Census and Survey Processing) software. This is a software package used for data gathering by the US Census bureau. |
| Assessment of database knowledge among a sample of data entry personnel/managers: |  |  |
| a. Could name the database | 1 | a. Data entry personnel/managers knew it is CSPro CAPI. However, the interviewers had no technical knowledge on how it worked. On the field, trained IT trouble shooters were available to assist with device and software glitches. Where further help was needed, the central office IT was contacted |
| b. Could describe what each database platform does | 1 | b. Data entry personnel/managers could describe the function of each database component |
| c. Know how to detect errors even before data entry by giving an example of such an error | 1 | c. Data entry personnel/managers were knowledgeable about errors even before data entry. They were able to offer examples of such errors, and what they needed to do when these errors were encountered. |
| Any justification for the selection of the database? | 1 | A major justification for this choice was that the software is free and publically available to end-users. The CSPro also combines features of the Integrated Microcomputer Processing System (IMPS) and the Integrated System for Survey Analysis (ISSA). This combination allows the performance of major tasks related to large surveys (such as the NAISS) including the following processes: data entry, data editing, tabulation, data dissemination, statistical analysis and data capture control so that the software platform could be used as a complete processing system or as stand-alone modules. IMPS allows persons with little or no computer experience to contribute to the data processing operation. Another advantage using the CSPro software is that it could handle complex survey data and is hosted on the user’s server. For the NAIIS project, the flat files from CSPRO were transferred to Microsoft SQL server. There was a customized data dictionary for the automatic creation of relational databases from the captured information. The CSPRO software for the survey had a CAPI (Computer-Assisted Personal Interviewing) application that allows editing of responses and signaled errors, features that permit data quality enhancement. |
| What is the architecture of the database? Does the architecture of the database correspond to the working conceptual framework? | 1 | The conceptual framework was to have a database that would capture multi-generational data with hierarchical components. The in-house health informatics team decided on the components of the database taking into consideration the reality of the terrain where you could have several families within a household, many children linked to a mother, etc. hence, at the planning and design stage, the choice for an appropriate and optimal database configuration was driven by this conceptual need. |
| Degree of complexity: E.g., is it a two-generation database (e.g., mother, child, father captured and could be linked)? | 1 | Multi-generational database to capture individuals nested in families that were nested in households. |
| Presence of data audit system E.g., regular sampling of the data and assessment for errors | 1 | When data entry was completed in the fields, the information was automatically transferred electronically to the main server at the call center which was used as the nerve center of the entire project implementation processes. Data were then examined in batches or clusters of 28 records at a time, and each record was scanned by a trained call center auditor for completeness as well as to identify inconsistencies and gaps. These flaws were then re-checked and reconciled with field data entry operators. About 1,500 records were audited through a rigorous monitoring system on a daily basis by a team comprising 30 well-trained and specialized data entry auditors grouped into teams with each team working on a 12-hour shift per day including weekends. |
| Presence of in-built checks mechanism (e.g., rejection of character where numerical values are specified and vice versa) | 1 | Integrated in the software was an in-built ***logic*** function that provided information consistency checks, and generated complex patterns and validation algorithms. For instance, the question on ART uptake could not precede HIV positivity date. This would trigger an in-built error alarm system to have the discrepancy corrected. |
| Presence of alert or inactivation system based on information non-concurrence (e.g., gender is male but the pregnancy space is filled out in the affirmative) | 1 | There were also in-built skip patterns (e.g., selecting the male option by the respondent inactivated all questions related to pregnancy status). |
| Presence of additional audit systems | 1 | In addition, periodic review of the forensic results provided additional audit avenues. |
| Does the database system in place employ a double key data entry validation process? | 1 | Certain questions related to study participants were cloned, and these questions were programmed and posed at three different survey phases of the data collection process. At the initial phase, the survey Team Lead collected data about household roster characteristics including socio-demographic features of household members to confirm household size and household member eligibility. This information was entered real-time by the Team Lead, and the data (on eligible persons) was delivered as a file to the individual-level interviewer using Bluetooth standard. The recipient interviewer would then conduct a fresh interview on each eligible household member using the roster. Integrated into the CSPRO-CAPI system was a self-check application that would now reconcile responses from similar questions already asked by the Team Lead. This audit step is, to some extent, analogous to the traditional double key data entry method. However, the addition of a third audit step rendered this method more robust. Completed data obtained by the individual-level interviewer were then sent (again using Bluetooth standard) to the field lab personnel for blood draw once informed consent was obtained. At this stage, some of the socio-demographic data pertaining to each individual were further verified and reconciled as needed, thus, providing a third key data entry point that completed the “***triple key data entry process***”. Before the end of the day, all gathered data were sent back to the Team Lead (Bluetooth) after completion and reconciliation. The Team Lead then forwarded the compiled information through a secure link to the server using FTPS (File Transfer Protocol Secure) connection to be delivered to the Central Office. |
| Was training provided for data entry/management personnel? | 1 | Prior to initiation of the study, a 3-week training session was organized for those that would be involved in the implementation of the survey including data entry personnel. The training was conducted in one place (Abuja) and by the same experts to maintain uniformity of training. The first day was dedicated to a pretest performed electronically to determine the baseline knowledge and skills of the course participants. At the end of the course, a post-test was offered and course attendees must have scored ≥ 90% (at least 90 out of 100) before they would qualify for field placement. Of the total number of 1,500 invitees, 1,330 were selected based on performance, qualification (at least a university degree) and other local language skills in addition to English language proficiency. |
| Is there a regular trouble-shooting session for the data entry/management team? | 1 | There were three levels for trouble-shooting that took place based on the degree of difficulty of the technical problem. The Team Lead on the field helped with mild-to-moderate IT issues that were within his/her competency. If the problem superseded the competency of the Team Lead, the designated IT specialist in the zonal office supervising the data collection processes for that area was called upon and, in most cases, the issue was resolved at this level. In less frequent cases, extremely unresolvable problems might arise with the data collection and entry activities requiring the attention of the more sophisticated Central Office where highly specialized IT personnel would be called upon to provide assistance. |
| Frequency of trouble-shooting sessions | 3 | Trouble-shooting sessions took place as they occurred, and supervision was immediately available at all three levels of supervision on a daily basis. |
| Is there any certification required for the data entry/management personnel before placement on the field? | 1 | Yes, attendees of the 3-week intensive training were conferred with a certificate of competency |
| Data-entry-sample ratio (DESR) = (the number of data management persons per 1000 samples per day). This is obtained by deriving the proportion of personnel per daily data waves. The greater the proportion the greater personnel adequacy and the lower the expected error rate. | 2 | There were 18 data entry persons that worked over the course of 150 days entering data from the field yielding a total of 2,700 data entry personnel days. On average, 1,380 individual interview records were entered per day from the 18 data entry personnel resulting in a total of 207,000 individual record entries over the entire course of the survey. Hence, the DESR = (18÷1380) × 1000 = 13 per 1000 per day. |
| Presence of a data management supervisor | 1 | There was at least one data management supervisor at each of the three levels of supervision (Team Lead; Zonal/sub-zonal coordinator; and at the Central Office in Abuja, the nation’s capital) |
| Presence of a data management deputy supervisor | 1 | A deputy was assigned in case the Team Lead was not available at the field level while a number of deputies were available in case the substantive supervisory head was unavailable at the Zonal and Central Office levels. |
| Qualification of Data Managers | 3 | Most of the data managers possessed qualifications beyond the first degree and the final average was closer to 3 than 2. |
| Qualification of Data Manager Supervisor | 3 | The minimum qualification for each data manager supervisor was a University degree. Most were holders of post-graduate degrees as well |
| Variable Missing Ratio (VMR) | 4 | There were a total of 1,817 variables in the dataset. Of these, only one variable had more than 10% missingness yielding a VMR of 0.0005 or 0.05%. |
| Observation Missing Ratio (OMR) | 4 | Of the 904,197 observations across five different questionnaires, there was not a single observation with more than 10% of the variable missing, yielding a OMR of 0/904,197 or 0%. |
| Duplicates ratio: total number of unique records divided by total number generated records. The lower the value below one, the higher the number of duplicates. | 1 | No duplicate records were possible due to the use of CAPI technology which automatically eliminated duplicates. |
| Presence of Data and Safety Monitoring Board (DSMB) to periodically evaluate accumulated data and ascertain data security | 1 | There was an overall data management subcommittee made of individuals that enjoy national and international reputation within their areas of expertise. They represented a huge professional resource to various aspects of the data management process. The subcommittee was comprised of statisticians, epidemiologists and public health experts from the Nigerian Federal Ministry of Health, UNAIDS, as well as methodologists from Nigerian Universities. The data management subcommittee provided overall guidance and supervision regarding data management issues, confidentiality of information and cybersecurity concerns. The body met about once monthly chaired by a prominent Nigerian academician, Professor EA Bamigboye. In addition to this supervisory body, the day-to-day database quality assurance activities were being undertaken by two specialized teams with ample knowledge of the database: 1) the data monitoring team; and 2) the data infrastructure integrity team. The data monitoring team was responsible for periodic auditing of each data point entered into the database to confirm its accuracy. In addition, it was also responsible for checking the completeness and appropriateness of audit trails and other data forensic diagnostics. Its overarching aim was to ascertain accuracy of the content of the database. The data infrastructure integrity team was concerned with data security including regular and frequent scanning to detect and address breeches or defects in the system. Hence, there were two levels of data management supervision: at the national level and at the facility level where physical data entry and management were being supervised. |
| If a form of DSMB exists, describe the membership and expertise of members of this or its equivalence | 3 | There were broadly two data management teams: (1) the data monitoring team; and 2) the data infrastructure integrity team. The data monitoring team was responsible for the accuracy and validity of data entry and statistical analyses. The team comprised statisticians, epidemiologists and physician scientists. The data infrastructure integrity team was responsible for the security and integrity of the entire database system including scanning for breaches and providing immediate remedies. Members of the data infrastructure integrity team consisted of computer scientists and computer engineers as well as other IT specialists. |
| Is there a database dictionary created? | 1 | Yes, there was a data dictionary being developed |
| Quality assessment of the database dictionary  a. Logical and coding language (e.g., child_age rather than age_2) | 1 | a. Appropriate and simplified recodes of the variables were created to facilitate variable name recognition. Exported formats in SAS were generated with coded and recoded variable definitions. A format is herein defined as a layout specification for how a variable is displayed or printed. |
| b. Each coded variable is described clearly and is self-explanatory | 1 | b. Each coded variable generated and exported in SAS was clearly defined. |
| c. Synonymous variables (those measuring the same attributes) are identified and explained | 1 | c. The formats of coded and recoded variables describing similar attributes in different ways were well-explained and characterized. |
| d. Value for each variable correctly coded and consistent (e.g., age in years throughout and NOT in years and months for some records) | 1 | d. There was consistency in variable value assignment across records. |
| e. User instructions on how to utilize the database/dataset are available | 1 | e. There was an ongoing plan to provide a data use manual to the general public as a guide on how to approach and analyze the data. As at the time of this report, the manual was at the developmental stage. |
| f. Database dictionary accessible online with a search function capability | 1 | f. The data use manual once completed would be made available online together with the dataset. |
| g. Available automatic updating system (e.g., modification of a variable in the database is automatically reflected in the dictionary) | 1 | g. The robust validation processes as well as the audit systems in place were re-assuring that subsequent modifications might not be needed once the data were made available to the public. Nonetheless, it was planned that where and when necessary, updates to the data would be made available on the national server that would host the dataset in the future. |
| h. Is there a blinded data generation and reconciliation mechanism in place (e.g., two anonymous statisticians produce results of a task independently and discrepancy addressed)? | 1 | h. There were primary and secondary reviewers for results generated from the database. After the primary reviewer created a code, two secondary reviewers examined the codes independently to ascertain accuracy and appropriateness. |
| *Concordance of prevalence estimates for selected variables and comparison with expected or projected estimates. | 5 | The following variables were utilized and their proportions in the survey were compared to those in the general Nigerian population (see Appendix A) : (i) Women age, classified as 15-24;25-29;30-39;35-39;40-64; (ii)Men age, classified as 15-24;25-29;30-39;35-39;40-64; and (iii) Place of residence, categorized as urban and rural. The respective Total Absolute Difference (TAD) was 8.69, 11.94, and 5.8. The Total Grand Absolute Difference (TGAD) was 26.43; and the Grand Absolute Mean Difference (GAMD) was 2.20 |
| Was a weighting algorithm considered | 1 | Yes, a detailed explanation on the process of computing the sampling weights were provided in the protocol as well as during the national presentation of the survey methodology. A database consisting of all sampled households and associated individuals along with all sampling parameters and selection probabilities was built to facilitate the calculation of all of the sampling weights required for analysis. |
| Justification of the weighting process is clearly stated or specified | 3 | Yes. The weighting process was based on sample selection probabilities that were derived using the two-stage cluster-sampling design. First, the selection probabilities were calculated separately for each sampling stage and for each unit of sampling. Then the overall selection probability of household within cluster of stratum was the product of the selection probabilities at the two stages of sampling. The sampling weights were then computed as the inverse of the corresponding selection probabilities. For household participants within the 15-64 age category, the household base weight was employed as the person-level base weight. Estimation of sampling errors took into account the complex features of the sample design such as stratification, clustering, and variable probabilities of selection. Ample justifications were provided for the adoption and implementation of these detailed procedures. |
| Appropriateness of the Weighting algorithm | 3 | Appropriate adjustments were made for: 1) children 14 years or younger because the corresponding person-level base weight was twice the household base weight because the sample of children was restricted to a random half-sample of the full household sample; 2) household and individual non-response; 3) HIV testing weights were also calculated separately because not all the interviewed respondents would agree to the HIV testing. |
| Presence of external independent monitors or database assessors that are not part of the stakeholders or any structure of the study | 1 | Yes, an independent US database management team developed the quality assurance instrument and did assess the validity of the database management system using the instrument. |
| Files backup and transfer systems | 1 | There was a file back-up system that was activated every 24 hours to archive files and folders for the purpose of restoration in case of an adverse event occurring to the data system. |
| Database Transparency Index | 2 | This was scored as 2 since data access was highly restricted as per protocol in order to preserve data integrity. |
| Database security and risk management procedures:  a. Prevention of unauthorized intrusion | 1 | a. For data security, multiple levels of access had been incorporated into the database security system. This included 1) a secure firewall that blocked untrusted or suspicious network traffic in order to prevent the likelihood of computer attacks that could compromise database integrity; 2) VPN (Virtual Private Network): this network device allowed a secure and privileged connection to access local network resources over the internet with enhanced security by preventing local resources and networks from direct internet exposure; 3) Password protection: database personnel could only gain access to certain information using authorized passwords. Information access was compartmentalized based on level of security clearance; 4) FTP (File Transfer Protocol) site: a standard network protocol was used for the transfer of computer files using separate control and data connections. For enhanced security of the network, only three individuals had access to the FTP site; and 5) Audit trails: which documented chronologically the sequence of events or changes that had been made to the database. |
| b. Audit trails of all activities by staff and administrators | 1 | b. There was an audit team dedicated to activities to document the history of events or procedures implemented in the database to enable, when needed, the reconstitution and review of the activity sequence as well as changes made to the database. An automatic suite of audit files was then generated including date, time, and user information associated with the procedure or transaction. |
| c. Database replication and synchronization: replication of database to other servers | 1 | c. There was a file back-up system activated every 24 hours to archive files and folders for the purpose of restoration in case of an adverse event occurring to the data system. |
| d. Well-defined compartments of privileges | 1 | d. Privileged access to the database compartments were stratified according to levels of security clearance so that only authorized database management personnel could have access to specific information or the ability to make changes to that information. |
| e. Coding review layering (how many verification layers ascertain accuracy of codes?) | 2 | e. There were three levels of coding. After the primary reviewer had created a code, two secondary reviewers examined the codes independently to ascertain accuracy and appropriateness. In case of discrepancy, a session of the primary and secondary reviewers was held to reconcile the differences and unanimously accept the correct code. |
| f. Periodic database vulnerability testing (e.g., automated vulnerability scans to uncover database defects): | 1 | f. As part of protocol compliance, periodic automated scans were performed to detect defects within the database. If any security defects or breeches were diagnosed, a specialized team within the IT unit would address them immediately. |
| g. Compliance monitoring to ascertain security standards are observed | 1 | g. As part of the overall comprehensive internal security program, compliance reviews including risk assessments, were implemented periodically to ascertain adherence to internal procedural rules and external regulations. |
| h. DAM (Database activity monitoring): e.g., through analysis of protocol traffic or observing local database activity on each server | 1 | h. Installed in the data system was a real-time database activity monitoring (DAM) system. The DAM application offered a comprehensive lens to the assigned DAM manager to visualize key performance indicators including traffic attributes. The emphasis was the detection of anomalies and their sources (e.g., IP addresses). A DAM log table provided a summary of the traffic activity monitoring processes. |
| i. Separation of duties between DAM and database administrators | 1 | i. A well-trained DAM expert was assigned, and the person worked independent of other database administrators. |
| j. A two-factor authentication system | 1 | j. There were two security barriers that needed to be overcome to access the database. The first was the VPN (Virtual Private Network) which allowed a secure and privileged connection to access local network resources over the internet using a unique password; and the second was the use of an authorized and different password to access the database itself. |
| k. Control system to prevent physical damage (e.g., from outage and extreme heat or power fluctuations) | 1 | k. The computers containing the database and database back-ups were protected against power surges and spikes using APC (American Power Conversion) protection devices. There was a comprehensive environmental protection system to shield the data from damage resulting from adverse climatic conditions such as overheating, poor air circulation as well as dust, debris, moisture and fire outbreak. |
